# Lab on a bead with oscillatory centrifugal microfluidics for fast and complete mixing enables fast and accurate biomedical assays

**DOI:** 10.1038/s41598-024-58720-5

**Published:** 2024-04-15

**Authors:** David E. Williams, Wei Li, Mithileshwari Chandrasekhar, Carsten Ma On Wong Corazza, Gerrit Sjoerd Deijs, Lionel Djoko, Bhavesh Govind, Ellen Jose, Yong Je Kwon, Tiffany Lowe, Anil Panchal, Gabrielle Reshef, Matheus J. T. Vargas, M. Cather Simpson

**Affiliations:** 1 Orbis Diagnostics Ltd, 14 West St, Eden Terrace, Auckland, 1010 New Zealand; 2 School of Chemical Sciences, University of Auckland, Private Bag 92019, Auckland, 1142 New Zealand

**Keywords:** Biomedical engineering, Analytical chemistry, Analytical biochemistry, Lab-on-a-chip

## Abstract

Rapid mixing and precise timing are key for accurate biomedical assay measurement, particularly when the result is determined as the rate of a reaction: for example rapid immunoassay in which the amount of captured target is kinetically determined; determination of the concentration of an enzyme or enzyme substrate; or as the final stage in any procedure that involves a capture reagent when an enzyme reaction is used as the indicator. Rapid mixing and precise timing are however difficult to achieve in point-of-care devices designed for small sample volumes and fast time to result. By using centrifugal microfluidics and transposing the reaction surface from a chamber to a single mm-scale bead we demonstrate an elegant and easily manufacturable solution. Reagents (which may be, for example, an enzyme, enzyme substrate, antibody or antigen) are immobilised on the surface of a single small bead (typically 1–2 mm in diameter) contained in a cylindrical reaction chamber subjected to periodically changing rotational accelerations which promote both mixing and uniform mass-transfer to the bead surface. The gradient of Euler force across the chamber resulting from rotational acceleration of the disc, d*Ω*_*disc*_*/*d*t*, drives circulation of fluid in the chamber. Oscillation of Euler force by oscillation of rotational acceleration with period, *T*, less than that of the hydrodynamic relaxation time of the fluid, folds the fluid streamlines. Movement of the bead in response to the fluid and the changing rotational acceleration provides a dynamically changing chamber shape, further folding and expanding the fluid. Bead rotation and translation driven by fluid flow and disc motion give uniformity of reaction over the surface. Critical parameters for mixing and reaction uniformity are the ratio of chamber radius to bead radius, *r*_*chamber*_*/r*_*bead*_, and the product *Tr*_*chamber*_*(*d*Ω*_*disc*_*/*d*t)*, of oscillation period and Euler force gradient across the fluid*.* We illustrate application of the concept using the reaction of horse radish peroxidase (HRP) immobilised on the bead surface with its substrate tetramethylbenzidine (TMB) in solution. Acceleration from rest to break a hydrophobic valve provided precise timing for TMB contact with the bead. Solution uniformity from reaction on the surface of the bead in volumes 20–50 uL was obtained in times of 2.5 s or less. Accurate measurement of the amount of surface-bound HRP by model fitting to the measured kinetics of colour development at 10 s intervals is demonstrated.

## Introduction

Rapid mixing and precise timing are critical for biomedical assays that rely on measurement of reaction kinetics. These considerations come to the fore particularly for point-of-care (POC) systems. Amongst the critical barriers to adoption are the accuracy of results, effect on clinical decision-making, patient experience, effects on work-flow and cost^1^ leading to accuracy, time-to-result, sample volume (finger-prick, for example) and simplicity of manufacture as important constraints. Measurements required^2,3^ include substances that can be measured through the rate of their enzymatic conversion (urea, creatinine, glucose are examples), critical diagnostic enzyme concentrations (alanine aminotransferase, an indicator of liver disease is one example) and a wide range of substances that are measured by immunoassay (antigen and antibody markers of infectious disease, cardiac markers TnI and BNP, and the inflammation marker CRP for example). Rapid immunoassay procedures that have been developed to address both time-to-result and sample-volume constraints illustrate the importance of reaction kinetics: they are operated in the regime where the amount of captured target is determined by the kinetics of the capture reaction and where the signal is determined by the kinetics of the signal-generating reaction. Lateral flow assays, where the target is incubated with nanoparticles as the capture surface whilst migrating along a porous support then through a detection zone, exemplify the issues^4^. Obtaining quantitatively accurate results in this regime requires accurate timing. Lateral flow systems suffer from variability in both the time scale for release of the nanoparticulate label into the fluid and in the migration rate of the fluid along the strip and through the detection zone. Variability in mixing of the signal-generating label into the sample results both in variable mass-transport and in heterogeneity of amount of captured target across the population of label particles. Whilst strips may be read using a simple reading device to quantify the development of colour on the capture zone, the uncertainty in timing and mixing severely limits the accuracy obtainable^4^. Similar considerations apply to the dry reagent strip chemistries that are used for a wide range of analytes^5^.

Microfluidic techniques^6^ offer the possibility to bring the timing under control, by accurately knowing when the reaction begins and when the measurement is taken. One critical issue however is achieving rapid and reliable mixing, of analyte fluids with diluents and reagents, in order to define the start time and ensure homogeneity throughout the reaction volume. Where reactions take place at a surface, rapid mass transport to the reaction surface is also required, in order to ensure that the system is surface reaction-rate controlled and uniform over the reaction surface area. Many methods have been proposed and extensively reviewed. Amongst the microfluidic methods, centrifugal microfluidics offers the substantial advantage of simple and precise control of the fluid flow, for timing accurately the beginning and end of the incubation steps, and of an extensive toolbox of structures that can be employed on the centrifugal disc for implementation of a range of necessary unit operations that can conveniently be strung together to give the required process flow^7^. However, as has been well-rehearsed in the literature, the small channel dimensions in microfluidic devices mean that flow is generally laminar. Mixing is then by diffusion only, and so can be slow. Stretching, splitting and folding of the flow is needed^8,9^. Centrifugal microfluidics offers a variety of methods to introduce vortical flow and chaotic eddies in order to increase the speed of mixing^9^. For fluids moving through channels, recent developments include variations on obstructions in the channel^10,11^, cross-flow systems^12,13^, side-chambers on the channel^14^, and variations in channel shape and cross-section^15–20^.

Implementation of rapid assay systems usually involves reaction of species in solution with other species adsorbed to a surface, which is typically within a chamber. To induce mixing within a chamber and uniformity of reaction with a surface, different strategies are required. Burger et al.^21^ described the combination of magnetic force with centrifugal fluidics. They induced reciprocating motion of an elastomeric membrane by integrating a permanent magnet on the membrane and using the oscillating magnetic interaction with a stationary magnet aligned along the orbit of the disc. They used this in conjunction with a system using trapped single beads to implement a multiplexed immunoassay^22^. Noroozi et al. described mixing in a reciprocating centrifugal microfluidic system, and the use of such a system to implement immunoassay^8,23^. A reservoir for storing pneumatic pressure was incorporated; centrifugal force and the resulting accumulated pneumatic pressure were used to propel two fluids to be mixed into and out of a mixing chamber, back and forth as the disc oscillated^8^. Complete mixing was achieved in less than 3 min. To implement an immunoassay, capture surfaces were deposited on the walls and the reciprocating rotational motion caused the analyte to move back and forth across these^23^. Lin et al.^24^ described a system for sample-to-result for blood coagulation tests. These measurements also require precise timing, on the scale of 1 s, so require rapid and repeatable mixing. They used large-amplitude rotational oscillation of a centrifugal microfluidic disc, essentially inducing a rapid ‘sloshing’ motion of the fluid in a partially-filled shield-shaped chamber. In this geometry, there was a significant effect due to the free liquid surface confined within the chamber. Grumann et al.^25^ described two mixing concepts for centrifugal microfluidics. In the first, magnetic beads within a chamber were periodically deflected as the chamber rotated past a fixed magnet. In the second, mixing was induced by periodic changes in the sense of rotation of the disc, with constant acceleration. Diffusive mixing within the 25 μL cylindrical chamber had a time-scale of 7 min; the vortices induced as a consequence of the periodic reversal of angular acceleration reduced the mixing time to ~ 3 s; mixing induced by the magnetic bead motion had a timescale of 1.3 s and a combination of the two strategies—bead motion and periodic reversal of angular acceleration—gave complete mixing with a timescale of 0.5 s.

Oscillating flows are easily produced in centrifugal systems by oscillating the rotation direction or by imposing a rotational acceleration that periodically changes. Amplitude and frequency of the modulation are control parameters. In order to obtain mixing within a chamber, this is attractive as a strategy that could induce the necessary flows in a structure that is easily manufacturable. Ren and Leung presented both numerical and experimental investigations^26,27^ describing mixing due to oscillatory rotation in a confined wedge-shaped chamber on a centrifugal microfluidic disc, where the angular acceleration followed a square-wave profile with time so the angular velocity had a triangular waveform. The Euler force due to the angular acceleration of the disc, being different at the inner and outer radius of the chamber, resulted in a primary vortex circulating around the chamber in the plane of the disc whilst the Coriolis force, acting on the fluid circulation in the primary vortex, caused a pair of secondary vortices circulating perpendicular to the plane of the disc. The net motion is toroidal. Transition to deceleration caused the primary vortex to reverse in direction. Reversal of the rotation caused the secondary vortex to reverse in direction. The resultant folding of the flow induced rapid mixing of two initially separated liquids within the chamber. In line with expections concerning the development of vortices and in line with numerical modelling, mixing times, typically 120 s for a chamber of volume approx 12 μL, decreased with increase of angular acceleration. The mixing time was shorter the larger the angular span of the chamber and the larger the ratio of outer to inner radial position of the chamber.

In the present work, we build on this literature and focus on speed of mixing and uniformity of transport to a reactive surface in relation to surface reaction rate in a model biomedical assay. The critical features are: the use of a single, large bead of near-neutral buoyancy which is the reactive surface, deployed in a shaped analysis chamber; and periodic change of angular acceleration and direction of rotation which, in combination with the bead and chamber shape, gives rapid, uniform mixing and mass transfer to the bead surface in a system that is easy to manufacture. The objective is to establish the basis for fast and accurate POC measurement on a small sample volume of an enzyme or substrate concentration, either as a measurement desirable in itself or as the final amplification and measurement stage of an immunoassay. In this implementation, the enzyme is supported on the bead. As proof-of-concept, we demonstrate the accurate measurement of the kinetics of the reaction between horse radish peroxidase (HRP) and tetramethylbenzidine (TMB) as a model system. A simple hydrophobic capillary stop, overcome by increase of rotation rate, provides the valve to control the injection of reagent into the reaction chamber at a precisely determined time^28,29^. Continuous measurement of colour development during the signal-generation step and subsequent curve-fitting to a reaction model both demonstrates the uniformity of mixing and reaction and improves both the precision and dynamic range of the measurement.

## Methods

**Figure 1:**
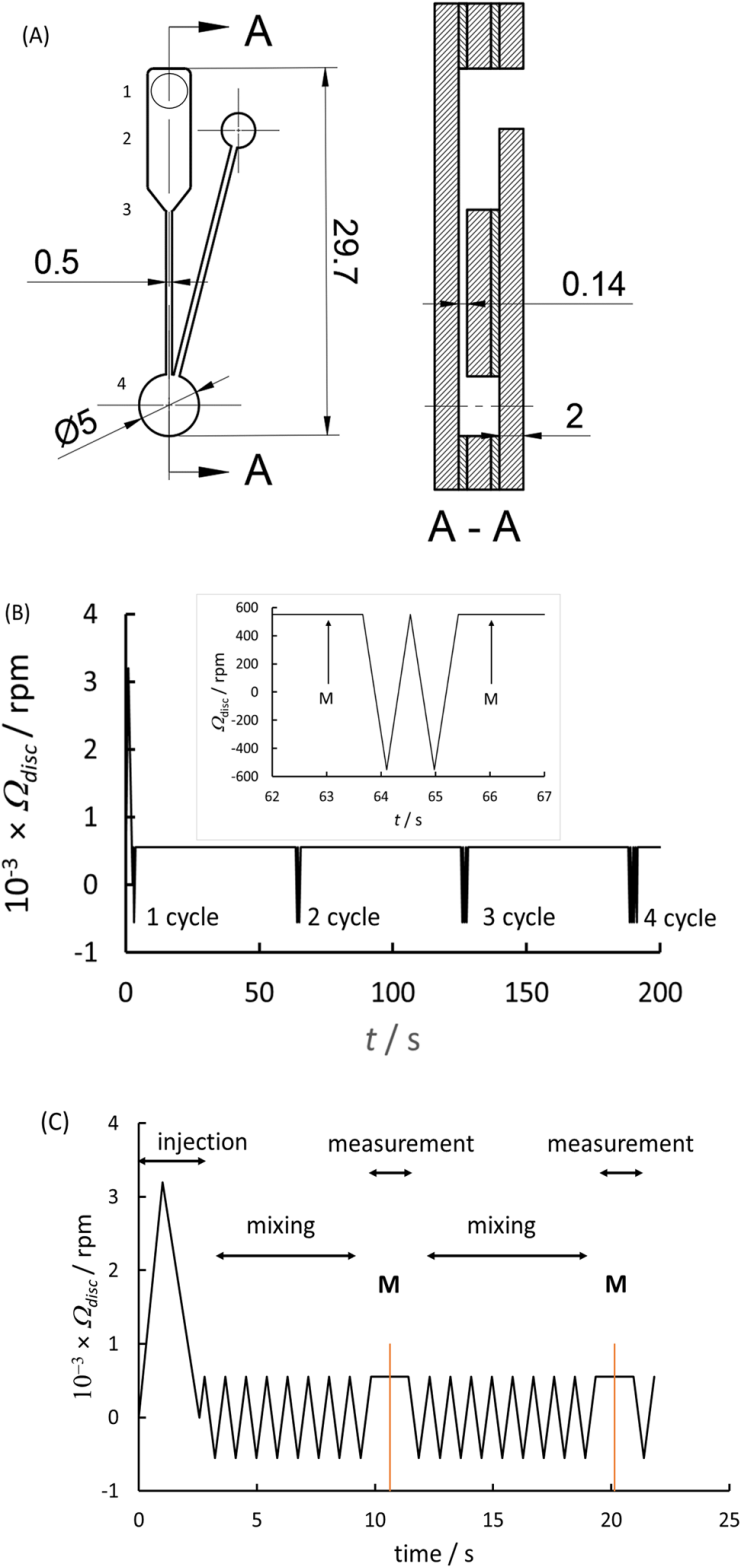
Disc assembly and rotation programme. (**A**) Inlet and reaction chamber in plan and cross-section along A–A, dimensions in mm; 9 of these were arranged circumferentially around the centre of the disc. The thin layers are double-sided adhesive tape through which the channels are cut and the thick are PMMA sheet through which the chambers are cut. (**B**) Rotation programme for exploration of mixing: rotation rate, *Ω*_*disc*_ (revolutions per minute) against time. The fluid, introduced through port 1 was held in the inlet chamber, 2 by the hydrophobic valve, 3^28^, then spun into the reaction chamber, 4, and mixed by periodic change of angular acceleration with intervals at constant rotation rate during which the optical transmission was measured (designated by M). After each measurement, mixing was paused for 1 min with the disc rotating at constant speed: hence the bead held at the radial position in the reaction chamber most distant from the disc centre. During this time, the solution would be static (no Euler force) and the blue reaction product would be being dispersed only by diffusion from the bead surface. The mixing cycle was then re-imposed following which the variation of optical transmission was measured again. (**C**) Rotation programme for enzyme kinetic measurement: following injection of TMB, the fluid was mixed by periodic change of angular acceleration with intervals at constant rotation rate during which the optical transmission was measured, at M.

*Discs and rotation programme*: Discs were laser-cut from poly(methylmethacrylate) (PMMA) sheet, 2 mm thick. The assembly comprised 3 layers, as shown in Fig. 1A. An image of the assembled disc is given in the supporting information (SI). The layers were bonded using double-sided pressure sensitive adhesive tape (Adhesives Research, product 90106NB). Chambers and through-valves (vents) were laser-cut through the central layer and the top layer, respectively. Channels connecting the chambers were formed by precision cutting the adhesive tape. The mixing chamber was cylindrical with radius 3–6 mm. The radial position of the centre of the reaction chamber was 45.6 mm from the centre of the disc. All surfaces were coated with a hydrophobic fluoropolymer (Cytopel 500, Cytonix LLC, MD, USA) before assembly. The channel entry at the base of the inlet chamber therefore formed a hydrophobic valve^28^, requiring a minimum pressure (applied by the centrifugal force) to move fluid from the inlet chamber into the channel. For imaging, a strobe light was synchronised with the motor drive. The motor was a brushless DC motor (Anahiem Automation BLWS232D) where speed was computer controlled and locked to the signal from a shaft encoder (4096 counts/revolution).

*Beads, and enzyme immobilisation*: Polystyrene beads of different diameter were obtained from Redhill Precision (Prague, CZ) and Cospheric (CA, USA). Beads were sensitised using passive adsorption of Goat anti-Human IgG Fc horse radish peroxidase (HRP: Thermofisher #A18817, RRID AB_2535594). HRP loading on beads was altered by change of concentration of the HRP adsorption solution. Antibody diluted from 1 mg/mL stock solution was shaken in the dark with 16 beads in a 4 mL glass vial for 1 h. The beads were washed three times then stored in buffer at 4 °C. Immediately before use, beads were removed from the buffer and dabbed dry with a tissue before insertion into the disc reaction chambers, after which the disc was closed with the top layer. The CV in the amount of enzyme adsorbed was separately assessed as 4.5%, by conventional measurement on a sample of 8 beads for each loading (128 in total) each in a sample well, and measurement of the colour intensity using a spectrophotometer following quenching the reaction with strong acid. The enzyme substrate, tetramethylbenzidine (TMB, with H_2_O_2_: Surmodics TMBW1000-01) with volume to exactly fill the reaction chamber was introduced through the inlet port 1 (Fig. 1a) and held in the entry chamber 2 by pinning of the contact line at the entry to the channel, 3.

*Mixing assessment*: The measurement sequence shown in Fig. 1B gave assessment of mixing into a fluid of a coloured reaction product generated at the surface of a bead. Increase of the angular velocity forced the TMB into the channel against the effect of surface tension^28^ and injected the fluid into the reaction chamber, 4. Video imaging showed TMB contacting the bead at *t* − *t*_*0*_ = 0.7 ± 0.1 s and that the chamber was full within ~ 1 s following the software command to increase rotation rate. The disc then rotated at a constant speed (550 rpm) for 1 min, to move the bead out of the laser beam path and allow the coloured reaction product to accumulate around the bead; optical absorption was measured. One cycle of acceleration-deceleration was then performed. Optical absorption was measured again. This cycle of pause, measure, mix and measure, was repeated with increasing number of mixing oscillations. Uniformity of solution composition was assessed by measuring the spatial variation of signal across the reaction chamber. Light from an intensity-stabilised red (650 nm) laser diode converged to a beam diameter of ≈ 0.04 mm at the reaction chamber then diverged to cover ~ 50% of the surface of the detection photodiode mounted above the chamber. The full range of measurement was 0–3000 counts. The signal was linear in light intensity over the full range. The photodiode signal was sampled at 230 kHz; 5 samples were taken and averaged for each trigger pulse from the shaft encoder. The average light intensity across the centre of the reaction chamber for a single rotation of the disc was determined for each chamber (14 measurement points).

*Kinetic measurement*: The measurement was initiated at *t* = *t*_*0*_ by initiation of disc rotation to pass the hydrophobic valve and inject the reagent into the reaction chamber: Fig. 1C. Mixing by oscillation of rotational acceleration and rotation direction was initiated at *t* − *t*_*0*_ = 2.6 s. For measurement, the disc rotation rate was held at 550 rpm, which moved the bead out of the laser path, and the measurement made during the period of constant rotation rate. The first measurement was made at *t* − *t*_*0*_ = 10.5 s (range 10.45–10.6 s) and thereafter at a fixed time interval of Δ*t* = 9.7 s (range 9.6–9.9) up to a total reaction time of 300 s.

*Computational fluid dynamics* used Ansys Fluent version 2023-R2.

## Results

### Assessment of mixing into the solution of material deposited within the chamber

**Figure 2:**
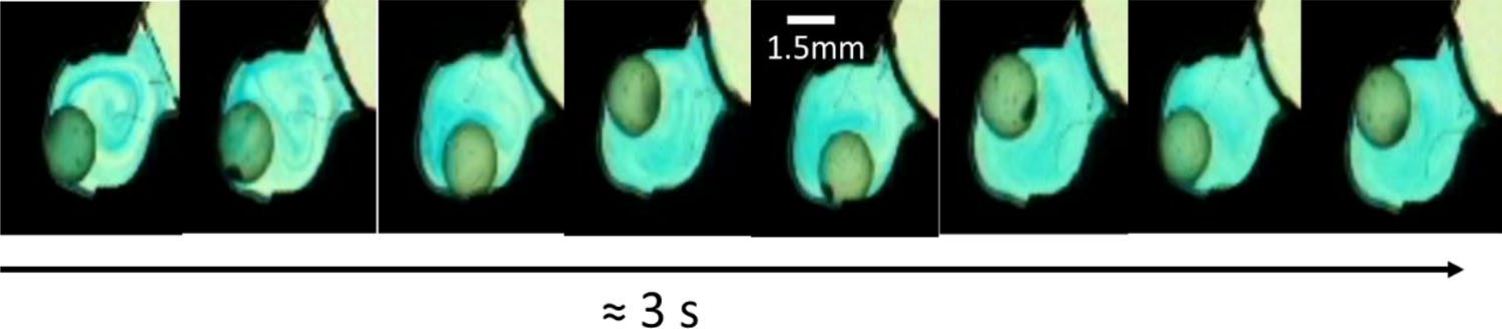
Frames from a stroboscopic video (Supplementary video 4) showing the progression of mixing after injection of clear solution into a chamber containing a small amount of dried dye together with a bead. Chamber diameter 3.1 mm, bead diameter 1.5 mm, d*Ω*/d*t* 2500 rpm/s, oscillation period 0.88 s.

The time scale for mixing induced by the oscillating flow and the oscillation of the bead in the chamber was first assessed by stroboscopic imaging. A small drop of dye solution was placed in the reaction chamber and dried with the bead before the disc was sealed. Clear solution was injected and the movement of the dye imaged during the injection and mixing. Figure 2 shows that mixing appeared complete within less than 5 cycles of oscillation of rotational acceleration of the disc (~ 4 s).

Bead density (buoyancy) or size in relation to the diameter of the chamber are important parameters. Stroboscopic videos showing the bead motion are given in the supporting information (SI: supplementary videos 1–3). Greater bead density or size in relation to the diameter of the chamber lead to the bead simply rolling around the periphery of the chamber in response to the reversal in angular acceleration of the disc. Smaller or more buoyant beads move through the middle of the chamber.

### Assessment of mixing into the solution of reaction product generated at the bead surface

**Figure 3:**
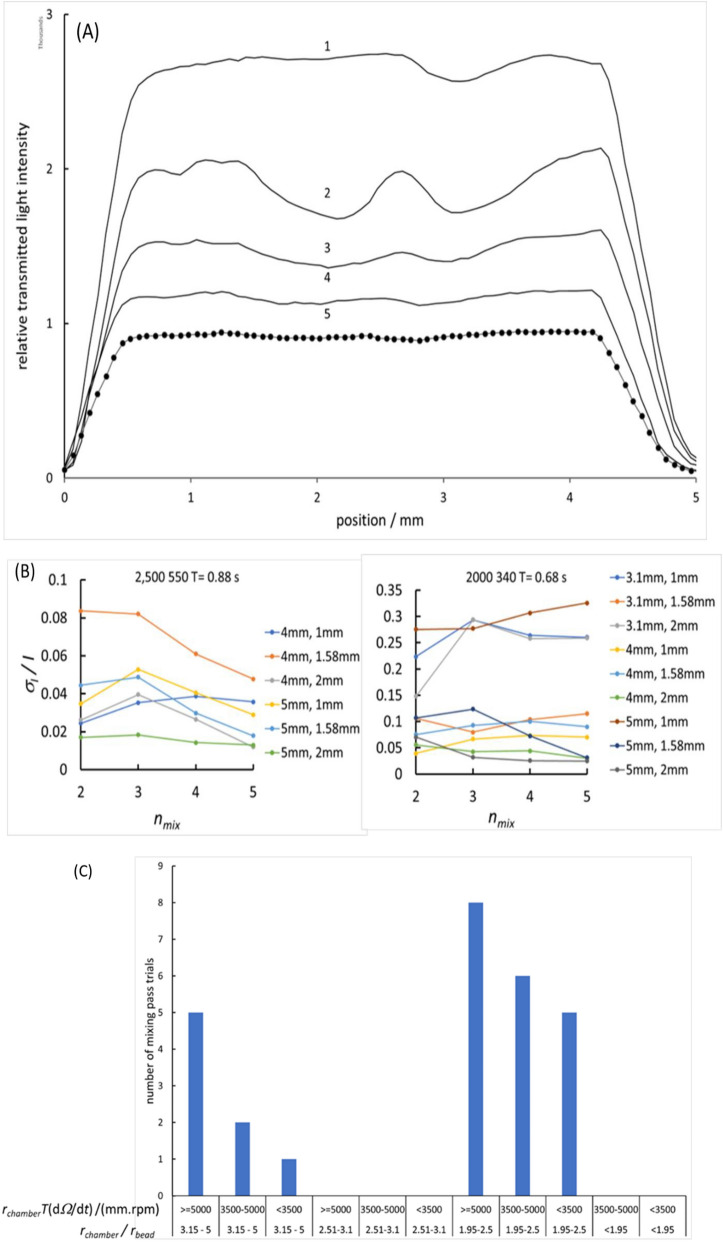
Mixing assessment during generation of coloured reaction product at the surface of the bead, by optical absorption scanning across the reaction well. (**A**) Development of solution uniformity with increasing number of cycles of disc angular velocity, following the procedure illustrated in Fig. 1B. Relative optical transmission in photodiode counts against position from the initial intersection of the laser beam with the edge of the reaction well. The curves are labelled with the number of oscillation cycles after each pause; 5 mm diameter reaction chamber, 1.58 mm diameter bead. $d{\Omega }_{disc}/dt$ = ± 2500 rpm/s; ${\Omega }_{disc,max}$= ± 550 rpm; disc oscillation period, *T* = 0.88 s; ${\Omega }_{disc,measurement}$= 550 rpm. (**B**) Example results illustrating the dependence of the progression of mixing on bead radius, chamber radius and rotational acceleration. Ratio ${\sigma }_{I}/\overline{I }$ of standard deviation to mean of optical transmission with position across the mixing chamber, on number of mixing cycles, *n*_*mix*_. Title: angular acceleration, maximum disc angular velocity, oscillation period; legend: chamber diameter, bead diameter. (**C**) Dependence of mixing on the ratio of chamber radius to bead radius, *r*_*chamber*_*/r*_*bead*_, and product *r*_*chamber*_*(*d*Ω*_*disc*_*/*d*t)*, with oscillation period of angular acceleration, *T*. Chambers of radius 3.1, 4 and 5 mm each with beads of 1, 1.58 and 2 mm diameter. A mixing ‘pass’ is determined as the standard deviation/mean of optical transmission on the laser path across the chamber less than an arbitrary threshold after 5 cycles. Total number of trials: 72; total number of ‘pass’: 33.

Mixing into the solution of reaction product from the surface of the bead was assessed by measurement of the variation of optical transmission as the reaction chamber moved past the laser beam, following injection of TMB into a chamber containing a bead with surface-adsorbed HRP. The procedure is shown in Fig. 1B. Figure 3A shows a typical result. With increasing number of cycles during the mixing phase, the solution composition, measured by the optical transmission, became more uniform.

The mixing time illustrated in Fig. 3 is 2.2 s. Effects of disc rotational acceleration, chamber radius and bead radius on this mixing timescale (5 cycles with cycle time 0.68–1.0 s) were evaluated by determining the ratio of standard deviation to mean of the optical transmission across the chamber as a measure of compositional uniformity. Figure 3B shows example results. All the parameters (bead radius, chamber radius, rotational acceleration, oscillation period) had an effect. Mixing tended to be more effective for larger chamber radius and for larger angular acceleration. A clear classification of effective conditions was obtained by examining the probability that the standard deviation/mean of optical transmission on the laser path across the chamber (Fig. 3B) was after 5 cycles less than an arbitrary threshold. For the range of oscillation period and rotational acceleration considered, the product ${r}_{chamber}T\left(d{\Omega }_{disc}/dt\right)$ is an approximate measure of the change of momentum of the fluid when the rotational acceleration (the Euler force driving the circulation) changes sign (see “Discussion”). Figure 3C shows that the larger this parameter, the more effective the mixing. There is also an interesting effect of the ratio *r*_*chamber*_*/r*_*bead*_: a clear optimum for mixing with *r*_*chamber*_*/r*_*bead*_ between 2 and 2.5 and a second range where mixing is improved when *r*_*chamber*_*/r*_*bead*_ > 3.1.

### Assessment of uniformity of reaction over the bead surface and uniformity of mixing by measurement of reaction kinetics

The measurement step for many types of bioassay is the determination of the amount of indicator reagent (in this case the enzyme HRP) bound onto a surface. In the “lab-on-a-bead” implementation, the bead is both the reaction surface and a means for promoting mixing in the solution. We demonstrated the accuracy of kinetic measurement enabled by precisely timed reagent injection and good mixing by determining the surface loading on the bead of the peroxidase enzyme. There are three methods for deriving this result from the measured signal, which all essentially estimate an approximation to the enzyme-loading dependent rate constant for colour development. The simplest is just to take the measurement at a specific time following the reagent contact with the enzyme-loaded bead. In the second method, the dynamic range of the assay is extended by using an empirical fit (eg polynomial) to derive the initial slope of the signal-time curve. In the third method^30^, the signal-time data are used to fit to a model for the reaction kinetics in order to derive the rate constant for the reaction—the method used here.

### Reaction kinetics and measurement model

The kinetics of the reaction of TMB with hydrogen peroxide catalysed by HRP are complex^31–33^. Denoting TMB as R, which is converted to the (yellow) di-imine product, P, with rate constant *k*, and the blue product as B, formed in a rapid equilibrium between R and P with equilibrium constant, *K*, the reaction can be formulated as: 1

Whilst a numerical solution is needed to solve fully for the concentration of the blue product as a function of time, for small conversion of TMB to product, the reaction model implies[P] *≈ kc*_*0*_*t* where *c*_*0*_ is the initial concentration of TMB. So

2 $$\left[ B \right] \cong \frac{{Kkc_{0} t}}{{1 + Kkc_{0} t}}$$

HRP shows Michaelis–Menton kinetics towards both H_2_O_2_ and TMB^32^ with *K*_*m,TMB*_ = 0.31 mM and *K*_*m,H2O2*_ ≈ 0.85 mM (pH 3.4). Commercial reagent preparations have concentrations of TMB and H_2_O_2_ sufficiently high that the rate constant,* k*, is essentially independent of the concentration of both TMB and H_2_O_2_ through the course of the reaction The reaction rate constant in Eq. (2)is therefore *k* = *k*_*E*_[E] where [E] denotes the concentration of enzyme in solution.

In the present realisation, the reaction occurs on the surface of a bead. The reaction flux at the surface (mole s^-1^) would be:

3 $$\frac{{dn_{P} }}{dt} = k_{s} S_{E} Ac_{0,s}$$

where *S*_*E*_ denotes the surface loading of enzyme (mole cm^−2^), *A* the surface area of the bead and *c*_*0,s*_ the concentration of reactant, R, at the surface of the bead. Hence the rate of increase of concentration of product, P, would be:

4 $$\frac{d\left[ P \right]}{{dt}} = \frac{{k_{s} Ac_{0,s} }}{V}S_{E}$$

where *V* denotes the volume of the reaction chamber. The bead is also the surface upon which the reaction takes place. Reaction of species from solution is characterised by a surface rate constant

5 $$k_s^{/} = \frac{{k_{s} }}{{\left( {1 + \left( {k_{s} /k_{m} } \right)} \right)}}$$

where *k*_*m*_ = *δ*_*D*_*/D* denotes the mass-transport rate constant (*D* is the diffusion coefficient of reactant and *δ*_*D*_ the Nernst diffusion boundary layer thickness) and *k*_*s*_ the surface reaction rate constant in the absence of any reactant concentration gradient. Then, taking into account the effect of mass transport of reactant, R, to the surface:

6 $$\frac{d\left[ P \right]}{{dt}} = \frac{{k_{s} Ac_{0} }}{V}\frac{1}{{\left( {1 + \left( {k_{s} /k_{m} } \right)} \right)}}S_{E}$$

The measurement made is of transmitted light intensity, *I*, (incident light intensity *I*_*0*_):

$ln\left(\frac{I}{{I}_{0}}\right)=-\varepsilon l\left[B\right]$ (*ε* is the extinction coefficient of the blue product and *l* the optical path length through the solution)

The transmitted light intensity would then be:

7 $$I = I_{0} exp\left[ { - a_{0} t/\left( {1 + a_{1} t} \right)} \right]$$

where

8 $$\begin{aligned} a_{0} & = \varepsilon \left[ \frac{l}{V} \right]\left[ {\frac{{Kk_{s} }}{{\left( {1 + \left( {k_{s} /k_{m} } \right)} \right)}}} \right]c_{0} \left[ {AS_{E} } \right] \\ a_{1} & = a_{0} /\varepsilon l \\ \end{aligned}$$

### Kinetic results and error analysis

**Figure 4:**
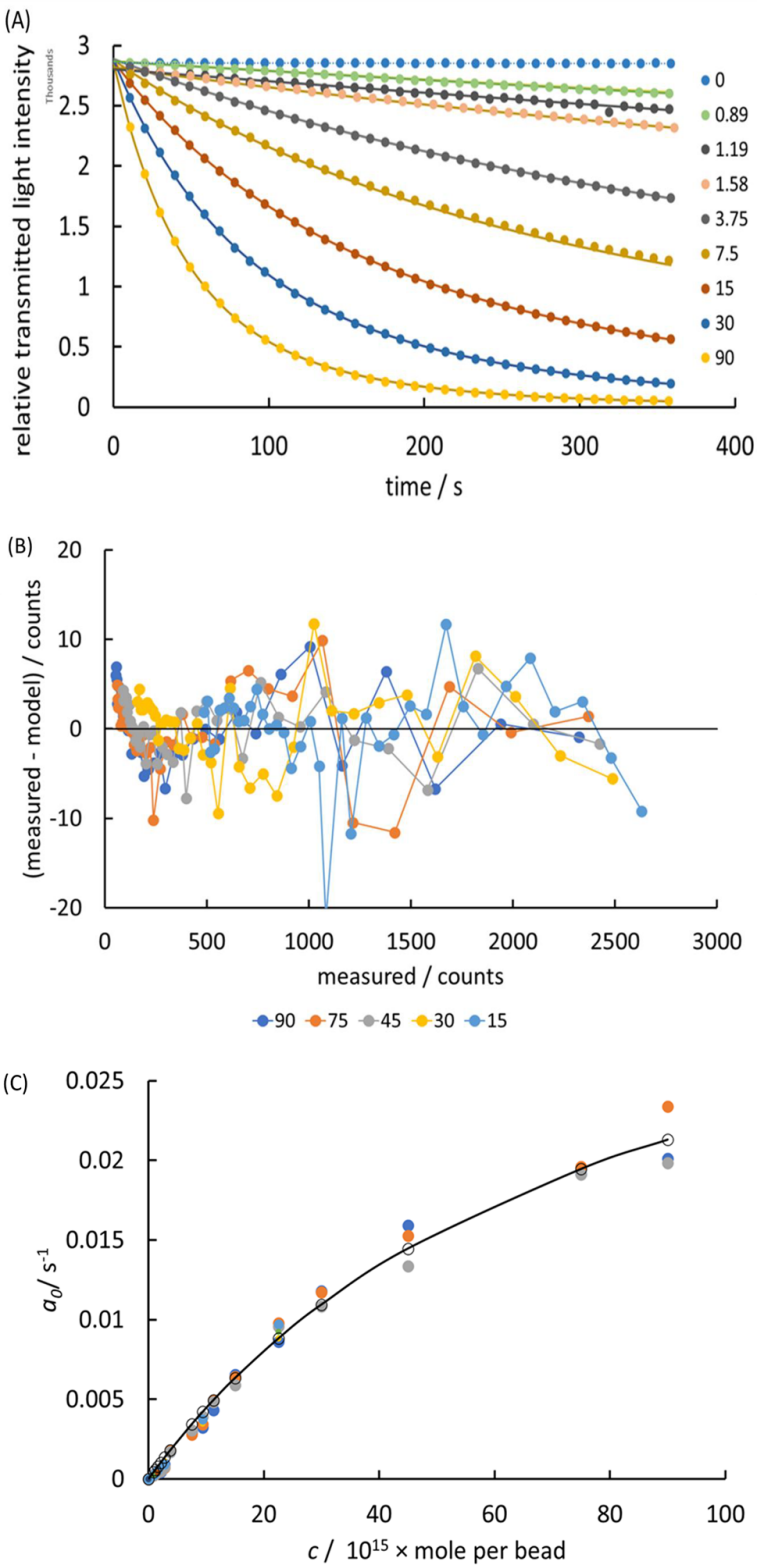
Assessment of mixing and mass-transport uniformity by determination of enzyme loading on a bead. Legends: 10^15^ × enzyme amount/mole per bead in adsorption solution (**A**) Variation of colour development with change of enzyme loading on the bead (points), illustrating the fit to Eq. (7)(solid lines). The rate constant extracted from the fit is the assay signal. Relative transmitted light intensity as photodiode counts/thousands against time/s. (**B**) Model bias variation: difference between measured and modelled photodiode counts with measured value. (**C**) Langmuir fit for enzyme adsorption: rate constant for colour development, *a*_0_ (Eq. 7) against enzyme concentration in adsorbing solution, expressed as mole/bead. Each point represents a different chamber and each colour a different disc.

A non-linear fit to Eq. (7)fitted the data well, over the experimental range. Figure 4A shows this fit. The fitting parameter *a*_*0*_ was taken as the experimental estimation of the desired result: the amount of adsorbed enzyme on the bead. The fitting error was estimated by adding zero-mean Gaussian random noise to both time and photodiode signal with standard deviation corresponding to the estimated variability in each and refitting multiple times. The coefficient of variation in *a*_*0*_ determined in this way was ~ 0.2%.

In order to assess the effectiveness of the timing of reagent injection and the mixing, and the potential impact on the precision of the result, the Bland–Altmann plot (Fig. 4B) shows the model bias variation. If the reagent composition was not uniform throughout the reaction chamber within a time scale that was short with respect to the rate at which the reaction was proceeding and short with respect to the time delay before the first measurement (10 s) then a systematic bias should be evident over the first few measurements. This is not observed, on the fastest reaction timescale studied (1/*a*_*0*_ ~ 50 s). Thus the reagent injection and composition uniformity was achieved on a timescale of order 5 s or less. The plot shows a small, systematic model bias developing at long time (greater extent of reaction) where the approximation behind Eq. (2) would be expected to fail. Figure 4C shows that the variation of *a*_*0*_ with concentration of enzyme in the sensitisation solution followed a Langmuir isotherm with some variability of results across different discs and chambers. The question arises: do these variations arise in variability due to inadequate mixing or non-uniform mass-transfer to the bead surface?

The objective is to assess the repeatability of the measurement across different chambers and discs in order to assess the contribution of different sources of variability in the experimental determination of *a*_*0*_, specifically in order to assess any variability that might be due to the mixing (homogeneity of the solution presented for measurement) and reaction uniformity over the bead. Sources of variability other than that due to mixing, timing and reaction variability over the bead surface are: first, the effect of variations in the chamber geometry, the factor $\frac{l}{V}$ in Eq. (8) As noted in the Methods section, the CV of this factor, determined by the fabrication procedure used in this case, would be ~ 9% (7% for chamber volume, *V*, and 5% for path length, *l*). The second source of variability is in the factor *AS*_*E*_, the total enzyme loading. The CV in total enzyme loading for the beads was ~ 4.5% giving a total CV of ~ 10%. Not accounted for is any effect of variation of temperature during the measurement giving a variation in rate constant.

For the different sensitising concentrations, across different bead preparations, discs and chambers, the observed coefficient of variation (standard deviation/mean) of *a*_*0*_ ranged from 1 to 16%. The weighted pooled coefficient of variation (72 measurements in total) was 13%. Thus, taking into account the variations in the geometrical factors and enzyme loading, the contribution to the coefficient of variation in the derived rate constant hence derived enzyme loading, due to variability in mixing and timing, and variation in the factor $\frac{K{k}_{s}}{\left(1+\left({k}_{s}/{k}_{m}\right)\right)}$ was less than about 3%.

## Discussion

The system has several features that aim to promote mixing through a complex, recirculating, strongly time-dependent flow. First, there are three forces (centrifugal, Euler and Coriolis) dependent on disc angular velocity and angular acceleration and fluid velocity that oscillate with the square-wave variation of rotational acceleration and with the triangular waveform of angular velocity with respect to time, and that act upon both the fluid and the bead [with magnitude dependent upon the bead buoyancy (${\rho }_{bead}-{\rho }_{fluid}$), *ρ* denoting the density]. Second, there is the time-scale for these oscillations, which is less than the hydrodynamic relaxation time for the fluid, ${\tau }_{H}={r}_{chamber}^{2}/\nu$ but also long enough that the length scale for damping of flow oscillations, $\delta \sim \sqrt{\nu T}$ is significant with respect to the length scales of the device (chamber radius and height, and bead radius). Here $\nu$ denotes the kinematic viscosity of the fluid (≈ 1 mm^2^ s^−1^ for water at 25 °C) so for the range of chamber radius and oscillation period used, ${\tau }_{H}=2.4-6.3$ s and *δ* ~ 0.5–1 mm. A significant momentum would be imparted to the fluid by the time the forces reverse. Third, the enclosed bead is a blunt body of size significant with respect to the chamber dimensions. The bead oscillates in position and rotates in response to the gradient of the oscillating forces, interrupting the flow and hence creating recirculating eddy flow behind it which must decay and reform following the abrupt motion caused by the reversal in sign of angular acceleration. The bead is also of near-neutral buoyancy, which means that it moves very fast in response to the acceleration of the fluid. The effect is to create a dynamically-changing chamber shape which forces reorganisation of the flow pattern with period *T*.

**Figure 5:**
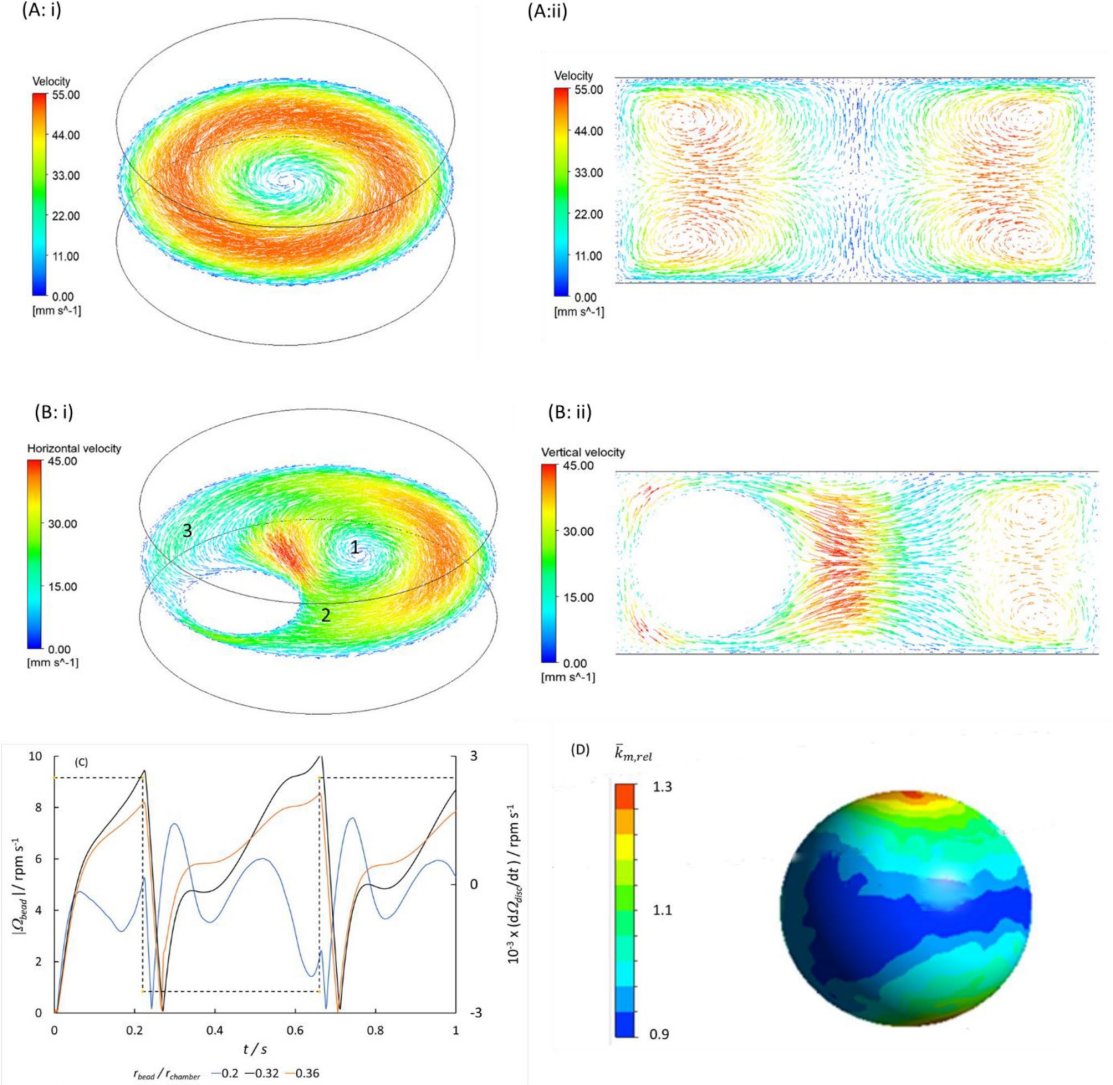
CFD of fluid motion in a cylindrical chamber subject to Euler force due to rotational acceleration. (**A**) Empty chamber; (**B**) with bead fixed in position, 0.2 mm from chamber wall: velocity vectors in planes through the rotation centre and centres of the chamber and bead (i) parallel and (ii) perpendicular to the plane of the disc rotation; *R* = 45.6 mm, *r*_*chambe*r_ = 2.5 mm, $d\Omega /dt$= 2500 rpm/s, *t* = 0.2 s after initiation of acceleration. Numbers on the different fluid zones refer to the text. (**C**) rotation rate of the bead through the cycle of rotational acceleration of the disc for different radius ratio *r*_*bead*_*/r*_*chamber*_. (**D**) computed time-averaged relative mass transfer coefficient, ${\overline{k} }_{m,rel}$ over the surface of the bead with the bead fixed in position but freely rotating.

Figure 3 indicates the importance of the parameter *Tr*_*chamber*_*(*d*Ω*_*disc*_*/*d*t)*. Computational fluid dynamics is helpful to understand the fluid motions and hence why this should be so. The Euler force, acting circumferentially, caused by the rotational acceleration, undergoing a square wave variation and reversing sign with period *T* has magnitude $\rho R\left(d{\Omega }_{disc}/dt\right)$: *ρ* is the fluid density, *R* the radial position with respect to the disc rotation centre and *Ω*_*disc*_ the disc angular velocity. The gradient of this force across the chamber, $\rho {r}_{chamber}\left(d\Omega /dt\right)$, with chamber radius,* r*_*chamber*_, drives a flow circulating in the plane of the disc around the cylindrical reaction chamber: Fig. 5Ai. The Coriolis force, acting upon the circulating flow induced by the Euler force, also oscillates in magnitude, changing sign as the primary flow changes sign and again as the direction of disc rotation reverses. The Coriolis force induces four symmetrically-placed secondary vortices rotating perpendicular to the plane of the disc: Fig. 5Aii. When the rotational acceleration changes sign, the forces change sign, so these flows decelerate then change direction; during this process, the flow streamlines fold over one another. In the absence of the bead, this process promotes mixing, as demonstrated by Ren and Leung^26^. Because the time scale for oscillation of the forces was shorter that the hydrodynamic relaxation time of the fluid motion, the fluid velocity varied with time throughout the periods at constant rotational acceleration (Fig. S2A,B) with short periods of rapid acceleration when the Euler force changed sign, and with the velocity gradient relaxing towards the centre of the chamber with time. The fluid velocity increased with cycle number (Fig. S2B). Approximately, the maximum fluid velocity, at the mid-radius of the chamber, is expected to be proportional to the torque acting on the fluid, $\rho {r}_{chamber}^{2}\left(d{\Omega }_{disc}/dt\right)$. CFD shows a linear variation over the experimental range of chamber radius explored (Fig. S2C). The velocities are on the same scale as those calculated by Ren and Leung^26^, for a wedge-shaped chamber.

The enclosed bead has a large effect on the flow within the chamber. The bead not only translates around the chamber wall in response both to the oscillating forces from the disc motion and to the fluid motion, but also spins in response to the gradient of fluid velocity^34,35^. As a consequence, the bead would also experience a lift force^36–38^, moving it away from the chamber wall. The resultant forces on the bead are dependent on the bead radius and would be opposed by any friction between the bead and the chamber wall, dependent on the bead buoyancy. In particular, the gradient of fluid velocity across the bead would depend not only on the chamber radius but also on the ratio of bead radius to chamber radius and on the bead position within the chamber. It would be expected to be maximum with *r*_*bead*_*/r*_*chamber*_ ~ 0.5.

In order to understand in part the effects, we used CFD for fluid motion around the bead with the bead fixed in position near the chamber wall. Figure 5B shows results. The bead divides the flow into a rotating zone (1), a wake or eddy zone (2) and an entry zone (3). Because the bead compresses the flow, the maximum velocity of the flow is decreased. We deduce that the ratio of chamber radius to bead radius is important in determining the flow pattern and flow velocity in the chamber. Whilst effective mixing might occur in the eddy zone away from the bead, other parts of the fluid would be less well-mixed if the bead were fixed in position. The motion of the bead is therefore a critical factor in securing uniform and effective mixing of the fluid. The fluid flow must decay and reform following the abrupt motion of the bead caused by the reversal in sign of angular acceleration. The reversal of direction of the flow displaces the rotating zone, and moves the eddy and entry zones from one side of the bead to the other. Further folding of the streamlines would result. However, if the bead simply rolled around the edge of the chamber in response to the disc motion, then depending on the radius of the bead in relation to the radius of the chamber there is the possibility that mixing of the fluid within and between the different zones is not promoted. Translational motion of the bead across the chamber would likely be important. A factor promoting that would be the lift force experienced by the bead. We explored using the Ansys CFD package how the angular velocity for rotation of the bead within the rotating frame of reference might change with changes with bead diameter, with the bead fixed in position and allowed to rotate only in the plane of the disc: Fig. 5C. The computed rotation rate varied through the cycle of disc rotational acceleration in a fashion consistent with the change of velocity gradient over time. The results are consistent with qualitative estimates from the literature^34,35^, with a complex dependence on *r*_*bead*_*/r*_*chamber*_. Finally, the non-uniform flow pattern across a fixed bead means that mass transport between components in solution and reagents on the surface of the bead would be very non uniform if the bead were fixed in position. Thus rotation and translation of the bead are important in securing uniformity of reaction over the surface of the bead averaged over time. We computed the mass transfer rate constant ${k}_{m}={\delta }_{D}/D$, where ${\delta }_{D}$ denotes the diffusion boundary layer thickness and *D* the solution species diffusion coefficient, for the bead fixed in position but allowed to rotate freely. Figure 5D shows that the computed value relative to the mean, averaged over time (10 s: ${\overline{k} }_{m,rel}$) varies by ~ 30% across the surface of the bead. Bead translational motion is likely to be important in further diminishing the variation.

The different zones of fluid motion illustrated by CFD (Fig. 5B) are evident in the stroboscopic images of dye mixing (Fig. 2). The importance of coupling deduced between bead and fluid motion is evident in the classification of mixing shown in Fig. 3. We can interpret the observation in terms of the coupling between bead and fluid motion, and the way in which the bead might constrain the fluid motion. Thus, in the range *r*_*chamber*_*/r*_*bead*_ between 2 and 2.5, the velocity gradient across the bead and hence bead rotation and lift force would be greatest, as indicated in Fig. 5. The effect would be to move the bead away from the chamber wall. Consistent with this interpretation of the importance of the fluid velocity gradient across the bead, beads that were either too large or somewhat smaller tended to roll around the chamber wall; the effect on mixing was diminished as a consequence as shown in Fig. 3 and anticipated by the flow patterns shown in Fig. 2. The movement of the smallest beads was again observed to be more throughout the chamber; the fluid velocity in the chamber with a smaller bead would be greater, which perhaps is the reason. The improvement in mixing in this size range is likely a result of that motion. Figure S2 illustrates that the fluid velocity increases approximately linearly with time, *r*_*chamber*_ and $d{\Omega }_{disc}/dt$. Hence the product *Tr*_*chamber*_*(*d*Ω*_*disc*_*/*d*t)* approximately represents the fluid velocity immediately before the Euler force changes sign: it is an approximate representation of the momentum change experienced by the fluid. Uniformity of reaction over the surface of the bead promoted by rotation and translation of the bead is affirmed by the study of reaction rate at a bead surface: the excellent fit to a reaction model and the minimal contribution to variability attributable to mixing in the system.

## Conclusion

The ‘lab on a bead’ is a powerful enabling concept for kinetics-based bioassay. A bead surface can be easily chemically modified and a range of reagents can simply be conjugated to it in precisely controlled ratios. Realisation of the possibilities requires also repeatable control of mass transport to the surface of the bead. Centrifugal microfluidics with constant angular acceleration gives a defined fluid velocity profile in an appropriately shaped chamber and also causes rapid rotation of the bead. Reversing the sign of the angular acceleration ensures movement of the bead through the bulk of the fluid. The resultant flow with uniform access to the entire surface of the bead gives the required control of mass transfer. Rapid mixing and homogenisation of composition of the fluid is also a requirement for aspects of kinetics-based assay systems, specifically for colorimetric measurement of a reaction product. Periodic reversal of sign of the angular acceleration causes a folding and reversal of the fluid flow. Movement of the bead periodically changes the shape of the chamber and causes reconfiguration of eddies in the fluid. These effects promote rapid homogenisation of fluid composition given particular ranges of ratio of chamber radius to bead radius, *r*_*chamber*_*/r*_*bead*_, and the product *Tr*_*chamber*_*(*d*Ω*_*disc*_*/*d*t)*, of oscillation period and gradient of Euler force. We have used a simple model assay system to demonstrate that the expected rapid and accurate measurement of enzyme loading on the surface of a bead can be realised in this way. The method can be applied quite generally in kinetics-based bioassay systems: for example, in immunoassay measurement of the amount of antigen captured onto an antibody-sensitised bead; or in coupled enzyme assays for either enzyme activity measurement or enzyme substrate measurement where the product of one enzyme reaction is the substrate for the next in a chain leading ultimately to a detectably coloured product. When the kinetics are well-defined because mass-transfer, solution homogenisation and timing are under control then systems do not need to go to equilibrium meaning that assays can be faster. Measurement of the time-course of product generation means that a reaction model can be used to fit the observed kinetics leading to a more reliable determination with wider dynamic range than can be achieved with a single time-point measurement.

### Supplementary Information

Supplementary Information 1.

Supplementary Information 2.

## Data Availability

The full set of data covering a range of disc rotational acceleration, chamber radius and bead radius is available from the corresponding authors on request.
