# Supplementary material for: Lab on a bead with oscillatory centrifugal microfluidics for fast and complete mixing enables fast and accurate biomedical assays
Source: Sci Rep. 2024 Apr 15;14:8637. doi: 10.1038/s41598-024-58720-5 (PMC11018808; doi:10.1038/s41598-024-58720-5)
Supplement: Supplementary file 1 — Supplementary Information 1. [file 41598_2024_58720_MOESM1_ESM.docx]

Supporting Information

**Lab on a bead with oscillatory centrifugal microfluidics for fast and complete mixing enables fast and accurate biomedical assays**

David E. Williams^,2^*, Wei Li ^1^, Mithileshwari Chandrasekhar ^1^, Carsten Ma On Wong Corazza ^1^, Gerrit Sjoerd Deijs ^1^, Lionel Djoko ^1^, Bhavesh Govind ^1^, Ellen Jose ^1^, Yong Je Kwon ^1^ , Tiffany Lowe ^1^, Anil Panchal ^1^, Gabrielle Reshef ^1^, Matheus J. T. Vargas ^1^ _,_ M. Cather Simpson ^1,2*^

^1^ Orbis Diagnostics Ltd, 14 West St, Eden Terrace, Auckland 1010, New Zealand

^2^ School of Chemical Sciences, University of Auckland, Private Bag 92019, Auckland 1142, New Zealand

*Corresponding authors. Email: [david.williams@auckland.ac.nz](mailto:david.williams@auckland.ac.nz) ; c.simpson@auckland.ac.nz

**Contents.**

Supplementary files

1. Supplementary videos 1 – 3. Stroboscopic imaging of the movement of beads of diameter 1, 1.5 and 2mm in a reaction chamber of diameter 3.2 mm with open neck. This reaction chamber design (Multi-chambered assay devices and associated methods, systems and apparatuses thereof for detection of analytes, MC Simpson, MJT Vargas, M Chandrasekhar, DE Williams, US Patent 11.565,260B2, 2023) is slightly more complex than the simple design treated in the main text in that, although the reaction chamber is cylindrical, it also has a section of free meniscus at the neck connecting to the inlet chamber. Images are captured as the rotational acceleration changes (maximum disc angular velocity both clockwise and counter-clockwise) and as the disc angular velocity passes through zero.

Video 1: 1mm diameter bead. Video 2: 1.5 mm diameter bead. Video 3: 2 mm diameter bead.

1. Supplementary video 4. Bead mixing of dried dye deposited in chamber. Polystyrene bead diameter 1.5 mm

Supplementary figures

Figure S1: Photo of an assembled disc

Figure S2: CFD of fluid motion in an empty cylindrical chamber subject to Euler force due to rotational acceleration. Disc radius *R* = 45.6 mm, chamber radius *r_chambe_*_r_ = 2.5 mm (A) Velocity profile in the parallel plane (legend: time / s from start of rotational acceleration). (B) Magnitude of the maximum velocity through the centre of the chamber as a function of time from the start of the rotational acceleration (rotational acceleration waveform superimposed); ${d\Omega}/{dt}$= 2500 rpm/s . (C) Maximum velocity in the parallel plane through the centre of the chamber, *v_max_* as a function of chamber radius and rotational acceleration (legend: rpm/s) at time *t* = 0.6 second


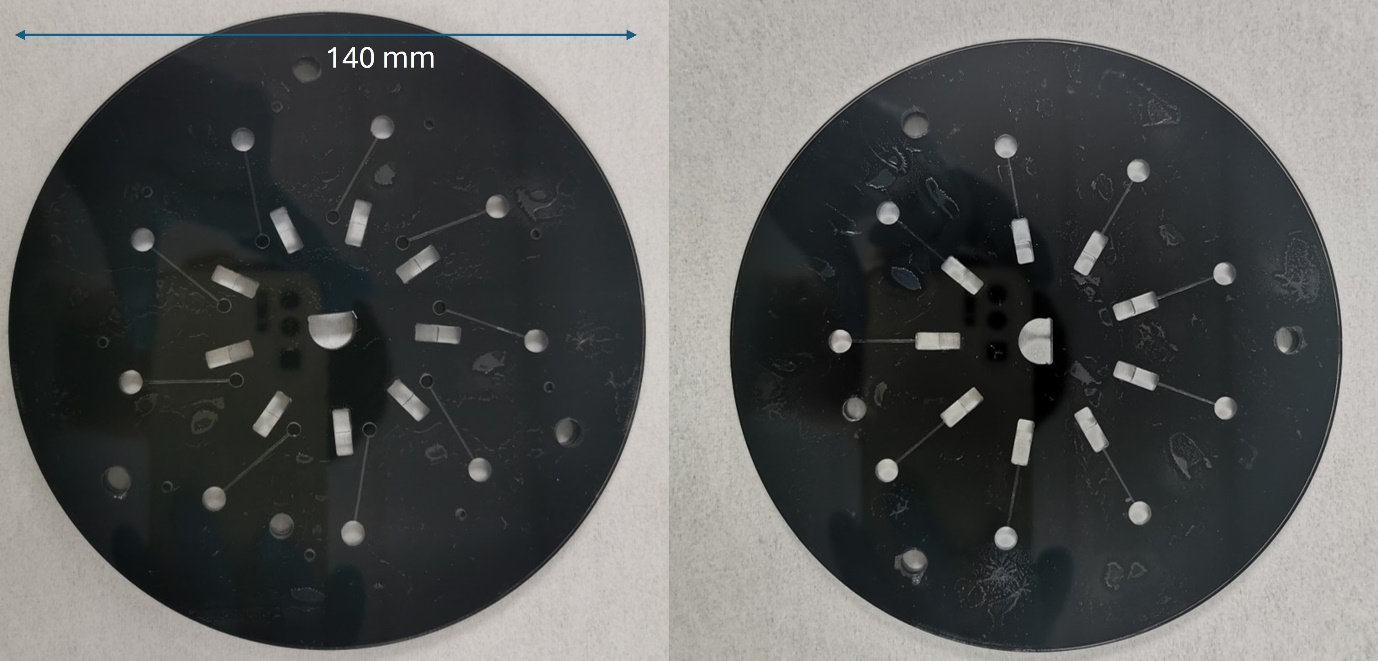


Figure S1


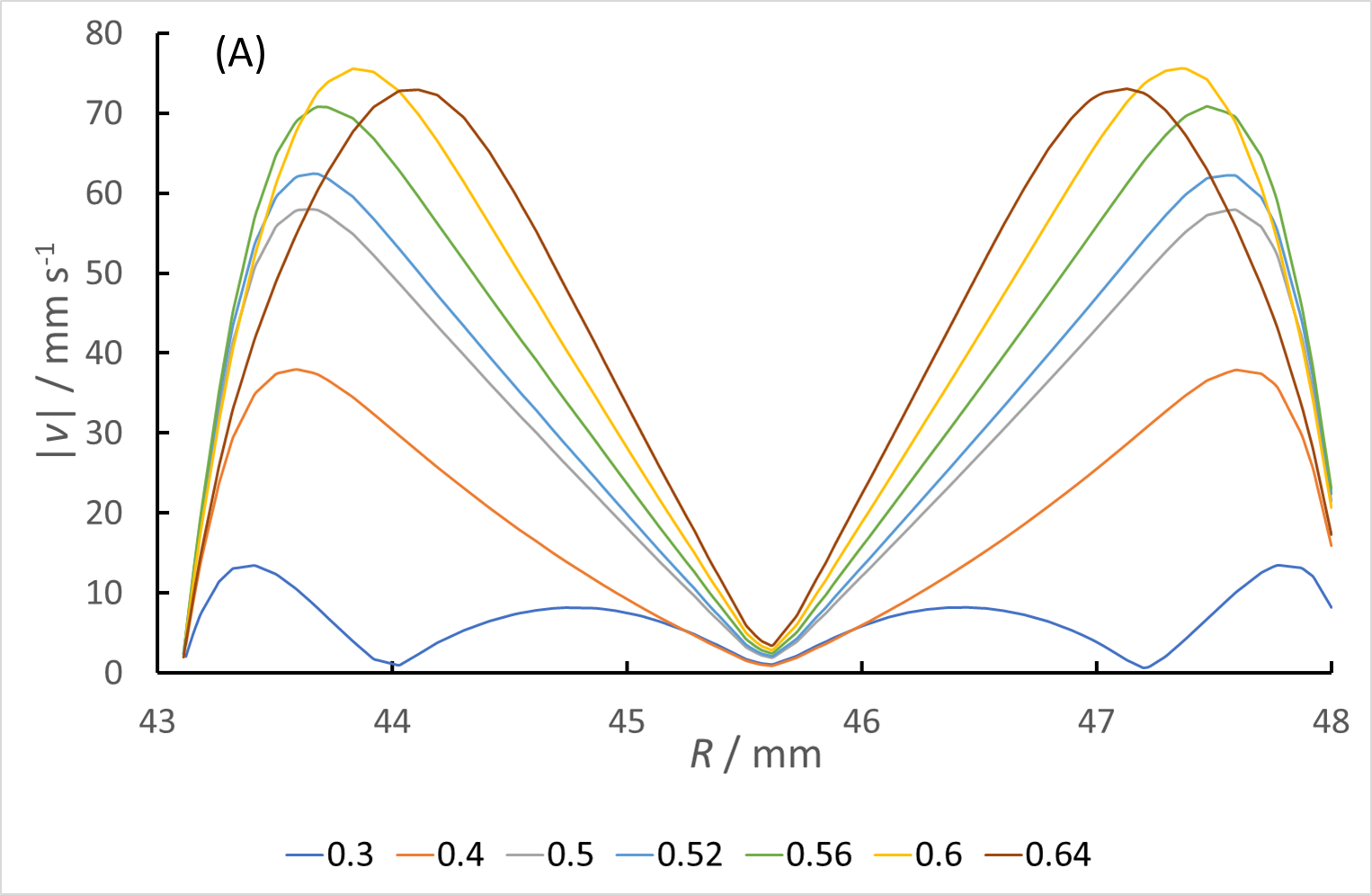


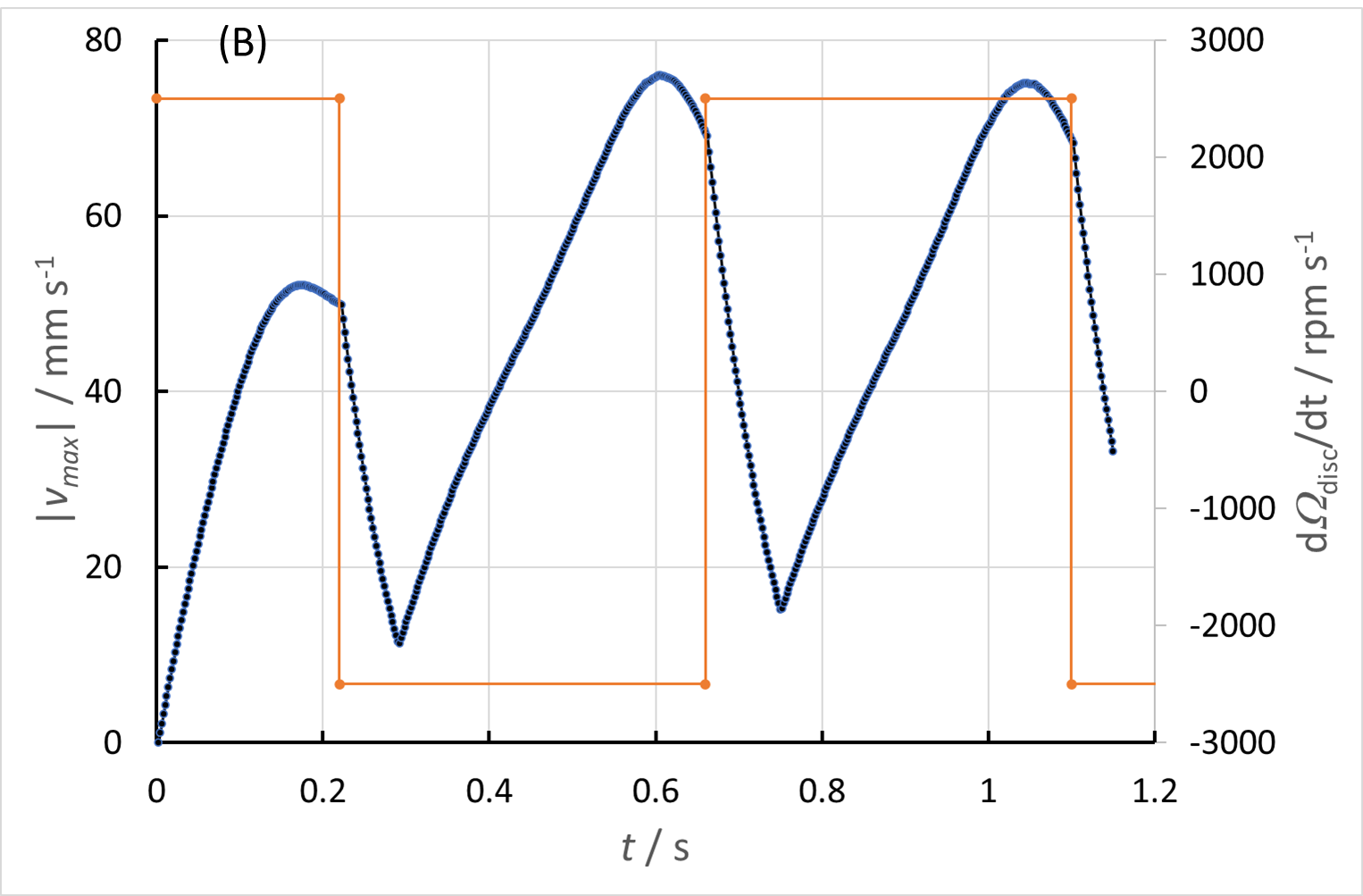


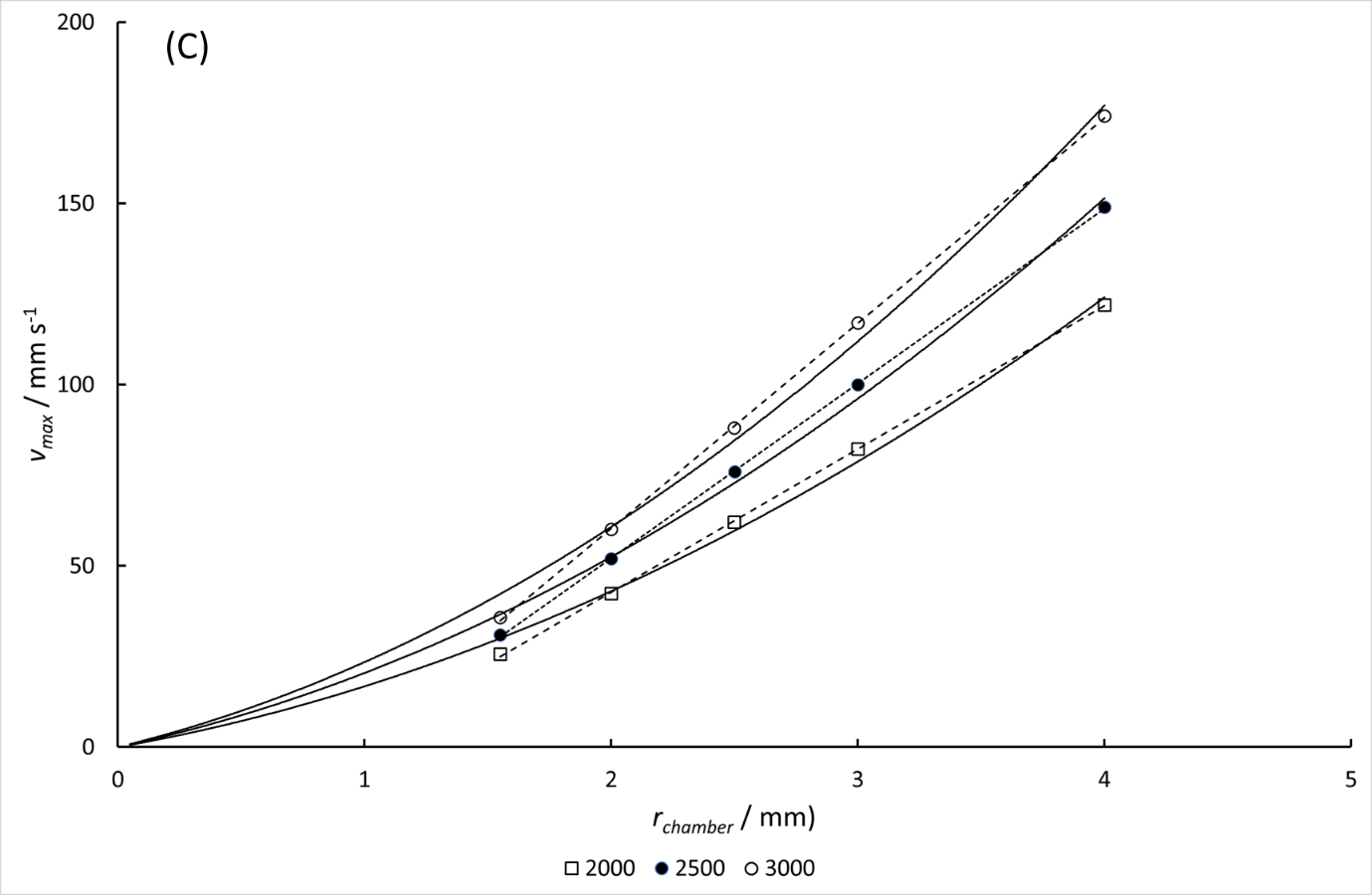


Figure S2
