# Supplementary material for: Lab on a bead with oscillatory centrifugal microfluidics for fast and complete mixing enables fast and accurate biomedical assays
Source: Sci Rep. 2024 Apr 15;14:8637. doi: 10.1038/s41598-024-58720-5 (PMC11018808; doi:10.1038/s41598-024-58720-5)
Supplement: Supplementary file 2 — Supplementary Information 2. [file 41598_2024_58720_MOESM2_ESM.zip › supplementary video 2 .zip!/supplementary video 2 caption.docx]

Supplementary video 2 . Stroboscopic imaging of the movement of a bead of diameter 1.5 mm in a reaction chamber of diameter 3.2 mm with open neck. This reaction chamber design ((Multi-chambered assay devices and associated methods, systems and apparatuses thereof for detection of analytes, MC Simpson, MJT Vargas, M Chandrasekhar, DE Williams US Patent 11,565,260, 2023 ) is slightly more complex than the simple design treated in the main text in that, although the reaction chamber is cylindrical, it also has a section of free meniscus at the neck connecting to the inlet chamber. Images are captured as the rotational acceleration changes (maximum disc angular velocity both clockwise and counter-clockwise) and as the disc angular velocity passes through zero. A smaller bead (diameter 0.3 mm) is also present to provide some indication of the fluid movement. A small trapped air bubble is also visible.
