# Supplementary material for: Lab on a bead with oscillatory centrifugal microfluidics for fast and complete mixing enables fast and accurate biomedical assays
Source: Sci Rep. 2024 Apr 15;14:8637. doi: 10.1038/s41598-024-58720-5 (PMC11018808; doi:10.1038/s41598-024-58720-5)
Supplement: Supplementary file 2 — Supplementary Information 2. [file 41598_2024_58720_MOESM2_ESM.zip › supplementary video 4.zip!/supplementary video 4 caption.docx]

Supplementary video 4. Bead mixing of dried dye deposited in chamber. Polystyrene bead diameter 1.5 mm. 0 -1 s: injection of fluid into chamber at constant disc rotation rate; 1 – 2 s: acceleration of the disc induces rotational motion of the fluid and initial dissolution and dispersion of the dye; 2s on: oscillation of disc rotational acceleration causes bead movement and fluid mixing.
